# Insulin-like growth factor 1 as a diagnostic marker of progressive central precocious puberty: a prospective study

**DOI:** 10.1007/s00431-025-06276-5

**Published:** 2025-06-24

**Authors:** Ana Luísa Leite, Luís Filipe Azevedo, Rosa Arménia Campos, Maria Adriana Rangel, Clara Torres, Filipa Marques Santos, Sónia Aires, João Firmino-Machado, Catarina Limbert

**Affiliations:** 1 Unit of Paediatric Endocrinology and Diabetes, Department of Paediatrics, Unidade Local de Saúde Gaia e Espinho, Vila Nova de Gaia, Portugal; 2 Clínica CUF, São João da Madeira, Portugal; 3 Department of Medical Sciences, University of Aveiro, Aveiro, Portugal; 4 Department of Community Medicine, Information and Health Decision Sciences (MEDCIDS), Faculty of Medicine, University of Porto, Porto, Portugal; 5 CINTESIS@RISE – Center for Health Technology and Services Research (CINTESIS) & Health Research Network Associated Laboratory (RISE), University of Porto, Porto, Portugal; 6 Department of Pathology, Unidade Local de Saúde Gaia E Espinho, Vila Nova de Gaia, Portugal; 7 Department of Radiology, Unidade Local de Saúde Gaia E Espinho, Vila Nova de Gaia, Portugal; 8 Department of Paediatrics, Unidade Local de Entre Douro E Vouga, Santa Maria da Feira, Portugal; 9 Unit of Paediatric Endocrinology and Diabetes, Hospital Dona Estefânia, Lisbon, Portugal; 10 Comprehensive Health Research Centre (CHRC), NOVA Medical School, NOVA University of Lisbon, Lisbon, Portugal; 11 Faculty of Medicine, University of Porto, Porto, Portugal

**Keywords:** Central precocious puberty, Insulin-like growth factor 1, IGF1-SDS

## Abstract

Central precocious puberty (CPP) diagnosis often requires invasive GnRH stimulation tests. Our purpose was to determine whether the IGF-1 and IGF-1 SDSs are reliable predictors of progressive CPP. This was a prospective study including 82 girls under 8 years of age. The participants were divided into CPP (*n* = 39), NP-CPP and IT (*n* = 26), and control groups (*n* = 17). Anthropometric measurements, Tanner staging, bone age, pelvic ultrasound, and serum IGF1 and IGF1-SDS level measurements were performed. GnRH stimulation tests confirmed CPP cases. The mean IGF1 and IGF1-SDS levels were significantly greater in CPP patients (270.15 ng/mL; 1.943 SDS) than in NP-CPP patients (174.12 ng/mL; 0.788 SDS) and controls (139.28 ng/mL; 0.208 SDS) (*p* < 0.001). Multivariate logistic regression analysis confirmed that IGF1 (OR = 1.025, 95% CI 1.010–1.040) and IGF1-SDS (OR = 8.721, 95% CI 2.624–28.986) were significant predictors of CPP. ROC analysis revealed an AUC of 0.837 for IGF1 (95% CI 0.738–0.935) and 0.862 for IGF1–SDS (95% CI 0.771–0.953). The cut-off values of 231 ng/mL for IGF1 (71.8% sensitivity, 97.7% specificity) and 1.44 for IGF1-SDS (79.5% sensitivity, 90.7% specificity) demonstrated good accuracy (82.2% and 77.8%, respectively). *Conclusion*: IGF1-SDS, and absolute IGF1, are promising effective noninvasive diagnostic markers for distinguishing CPP from nonprogressive precocious puberty. Due to its high specificity IGF1 values above 1.44, SDS may significantly increase the post-test probability of CPP, potentially avoiding invasive GnRH stimulation tests. These findings support the integration of IGF1 measurements into the initial diagnostic approach for girls presenting with early pubertal signs.

**Table:** 

**What is Known:** • Central precocious puberty (CPP) often requires invasive GnRH stimulation tests. • IGF1 levels rise during puberty and reflect growth and pubertal progression. **What is New:** • This prospective study suggests IGF1-SDS >1.44 as accurate cut-off for progressive CPP via the IMMULITE assay. • IGF1-SDS show high specificity and diagnostic accuracy, supporting its use in the initial diagnostic approach for girls presenting with early pubertal signs.

**What is Known:**

• Central precocious puberty (CPP) often requires invasive GnRH stimulation tests.

• IGF1 levels rise during puberty and reflect growth and pubertal progression.

**What is New:**

• This prospective study suggests IGF1-SDS >1.44 as accurate cut-off for progressive CPP via the IMMULITE assay.

• IGF1-SDS show high specificity and diagnostic accuracy, supporting its use in the initial diagnostic approach for girls presenting with early pubertal signs.

## Introduction

Puberty represents a dynamic phase of transformation, shifting from childhood to a fully mature adult form with complete reproductive capability [1]. Over the past century, there has been a notable trend of earlier onset of puberty, accompanied by an increase in the incidence of normal variants such as premature thelarche and cases of precocious puberty [1–3].

Precocious puberty is defined by the development of secondary sexual characteristics before the age of 8 years in girls and 9 years in boys [4]. It can result from distinct etiological mechanisms that influence the regulatory factors of pubertal timing, where genetic and epigenetic causes of CPP are novel areas of scientific investigation [5]. The most prevalent form of precocious puberty is central precocious puberty (CPP), characterized by the premature activation of pulsatile gonadotropin-releasing hormone (GnRH) secretion [6]. In addition to the early development of secondary sexual characteristics, patients with central precocious puberty (CPP) exhibit rapid linear growth and advanced skeletal maturation. Consequently, CPP mirrors the progression of normally timed puberty and, if left untreated, leads to early menarche or gonadarche, at which point minimal growth potential remains, often resulting in reduced adult height [7].

However, most girls exhibiting early pubertal development have common variants rather than pathological conditions requiring treatment. Therefore, distinguishing these incomplete or nonprogressive forms from complete forms is crucial to reassure patients and parents about the lack of pubertal progression and to avoid unnecessary treatment with LHRH analogues [8, 9]. This distinction has become increasingly challenging, especially since the COVID-19 pandemic, which has further elevated the global incidence of CPP [10–12].

The gold standard for diagnosing CPP is laboratory confirmation of hypothalamic‒pituitary‒gonadal (HPG) axis activation via stimulation testing with either GnRH or the synthetic GnRH analogue leuprolide, with a peak stimulated LH concentration greater than 5 U/L [6]. Nevertheless, it involves repeated blood sampling, which increases discomfort for children, places a financial burden on parents, and is challenging to perform in an outpatient setting, which explains why many researchers continue to look for other markers to find a simpler diagnostic method for CPP. Several of these markers, such as alfa-klotho or irisin, are only used experimentally, and others, such as inhibin B, are expensive for routine use [13–17].

Serum insulin-like growth factor 1 (IGF1) is a 70 amino acid polypeptide mitogen essential for growth that is very well known in paediatric endocrinology, particularly whenever there are growth concerns [18]. IGF1 increases slowly in early childhood, with an even greater increase during puberty. Serum IGF1 levels at puberty are in the acromegalic range of those in adults, with peak values occurring approximately 2 years after peak growth velocity in both sexes [19]. In healthy prepubertal children, IGF1 levels predict height velocity in the following year, which can be extremely valuable in the diagnosis of precocious puberty [18–21].

Our study aimed to evaluate the IGF1 and IGF1-SDS profiles in children with precocious puberty compared with controls to determine their potential as diagnostic markers for CPP, particularly in differentiating progressive forms from incomplete or nonprogressive forms. If the IGF-1 and IGF-1 SDS prove to be reliable predictors of progressive puberty, we will further assess the utility of measuring these levels during the initial screening visit, potentially reducing the need for unnecessary GnRH stimulation tests.

## Materials and methods

This observational prospective study enrolled patients from June 2022 to December 2024. This manuscript is written according to the Strengthening the Reporting of Observational Studies in Epidemiology (STROBE) guidelines and the Standards for Reporting Diagnostic Accuracy (STARD) guidelines [22, 23].

### Study population

Participants were recruited from tertiary- and secondary-level hospitals in the northern area of Portugal.

The study included two cohorts: girls with clinical signs of puberty from outpatient clinics and a control group of prepubertal girls from orthopaedics and surgery clinics. Only girls were included because central precocious puberty (CPP) is rare in boys, making recruitment difficult in this prospective study.

The sample size was calculated to detect differences between the means of the IGF1 levels of the compared groups. We used methods, formulas, and algorithms available at https://sample-size.net/. A significance level of 5% (*α* = 0.05) and a power of 80% were used. The effect size was based on data from previous studies reporting a mean difference in IGF1 levels of 120 ± 100 ng/mL [24]. Accordingly, the estimated sample size required was 26 individuals (13 per group) for IGF1. Having recruited 82 participants, 39 of whom had disease (progressive CPP), as defined by the gold standard, we were able to estimate the index test sensitivity and specificity, respectively, with margins of error (half-widths of the 95% confidence intervals) of 14% and 11%.

**Fig. 1:**
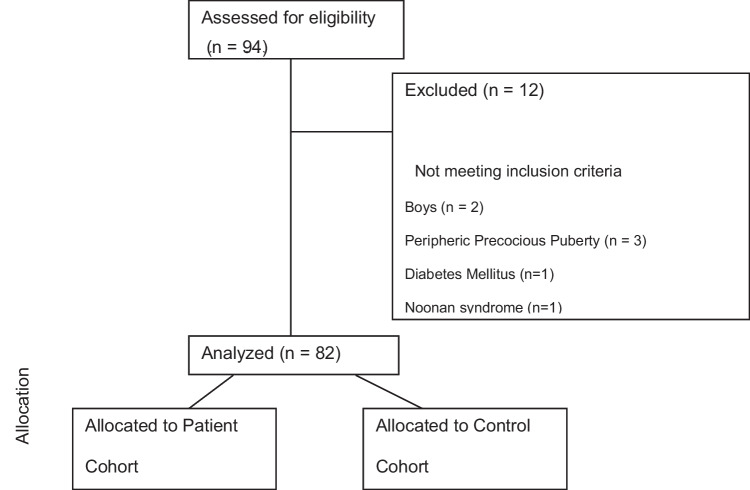
Participant flowchart

Eligible participants had to be female and 8 years old or younger. Among the 94 participants, 82 girls were included (Fig. 1). The exclusion criteria included peripheric precocious puberty, a previous history of brain tumours, cranial radiation, neurologic symptoms, genetic syndrome, hypopituitarism, diabetes mellitus, or chronic therapy with somatropin, insulin, or oral steroids.

### Participant evaluation and classification

All girls were classified as having pubertal signs when at least their breast development was present before 8 years of age (*n* = 65). The controls were all prepubertal (*n* = 17).

The central precocious puberty (CPP) definition included the following [6, 25]: breast development in girls younger than 8 years, typically with concurrent acceleration in growth velocity and bone age advancement, and laboratory confirmation of HPG activation by stimulation testing via GnRH stimulation with a peak stimulated LH concentration greater than 5 IU/L.

Inclusion in nonprogressive CPP (NP-CPP) occurred whenever the Tanner stage did not progress during follow-up and height-SDS remained similar. Isolated premature thelarche (IT) was analysed together with nonprogressive CPP.

After providing informed consent, all the girls were evaluated at Unidade Local de Saúde Gaia e Espinho. The physical examination and Tanner stage [26] were assessed by the same physician, who was blinded to the laboratory and radiological results.

Anthropometric assessments were conducted with the patients in underwear: weight was measured via a Seca™ scale (Mod 220, sensitive for 0.1 kg; Seca, Hamburg, Germany), and height was measured via a Seca™ wall-mounted stadiometer (Mod 240, Seca). Body mass index [BMI = weight (kg) ⁄ height^2^(m)] and standard deviation scores (SDSs) from auxologic measures were calculated via AnthroCalc™ 2.7.2 considering World Health Organization references. Mid parental height was calculated as [(mother height + father height) – 6.5 cm]. Bone age was measured by two blinded physicians via the Greulich and Pyle atlas [27]. When the results were discordant, a third examiner evaluated the data. The predicted adult height was calculated via the Bayley and Pinneau tables [28].

All the children fasted for 8:00; blood samples were collected for glucose, liver, kidney, and thyroid function; and FSH, LH, estradiol, IGF1, and anti-Mullerian hormone (AMH) levels were measured. All laboratory measurements were performed in the same laboratory, and the laboratory investigator was blinded to the participant groups.

IGF1 was determined via the IMMULITE Siemens 2000™ chemiluminescent enzyme immunometric assay, and IGF1-SDS was assessed via the new standard IS 02/254.

The stimulating test was performed using 100 mg/m^2^ LHRH (Gonadorrelin Acetate, Ferring™, Germany) with blood samples collected at baseline and at 30, 60, 90, and 120 min after the iv bolus in all children with pubertal signs [6].

Every girl with clinical signs of puberty underwent a pelvic ultrasound, which was performed by the same clinician, to evaluate uterine size and proportions and ovary volume. The results were classified as pubertal or prepubertal according to the presence of any of the following parameters: (1) uterine longitudinal diameter ≥ 3.5 cm or transverse uterine diameter ≥ 1.5 cm, (2) presence of an endometrial echo, and (3) ovarian volume ≥ 2 cm^3^ [29, 30].

All girls had a 3- and 6-month follow-up and were classified as having progressive CPP when rapid sexual development was present, height velocity increased, or/and bone age advanced [15].

### Statistical analysis

Statistical analysis was performed via SPSS™ version 29.0 software (SPSS Inc., Chicago, IL, USA). All probabilities were two tailed, and *p* values < 0.05 were regarded as significant. The distributions of the quantitative variables are presented as the means ± standard deviations (SDs) and were compared via Student’s *t* test when normally distributed or via ANOVA, as appropriate. Bonferroni adjustment was chosen whenever necessary. Quantitative variables with a distribution different from the normal distribution, median, and interquartile range (IQR) were used and compared via the Mann‒Whitney test. Qualitative variables are described as counts and proportions and were compared via the chi-square test or Fisher’s exact test. To evaluate the independent effects of height-SDS and bone age on IGF1 and IGF1-SDS, multivariable logistic regression models were computed. Receiver operating characteristic (ROC) curves were generated to determine the sensitivity, specificity, accuracy, and Youden index, all with 95% confidence intervals (CIs). Finally, ROC curve analysis was used to identify the cut-off values of IGF1 and IGF1-SDS that best identify patients with progressive CPP.

## Results

In our cohort, 65 girls had pubertal signs, and 17 were age-matched controls. Among the 65 girls with pubertal signs, 39 (60%) met the criteria for progressive CPP. The clinical and radiological characteristics of the patients are summarized in Table 1.

**Table 1:** Clinical and anthropometric characteristics of the study population and comparisons between the group with pubertal signs and controls and between the progressive CPP group and the nonprogressive CPP (NP-CPP) group

Total sample = 82	Pubertal signs *n* = 65				Controls *n* = 17	*p value*
		Progressive CPP *n* = 39	NP-CPP *n* = 26	*p value*		
**Age** (*y)*	6.87 ± 1.67	6.94 ± 1.80	6.76 ± 1.48	0.666	7.15 ± 1.74	0.535
**Age of 1 st pubertal signs** *(y)*	5.67 ± 1.60	6.00 ± 1.23	5.16 ± 1.97	0.065	–	
**Weight** *(kg)*	29.17 ± 6.42	29.41 ± 6.59	28.81 ± 6.26	0.712	26.98 ± 7.82	0.235
**Weight-SDS**	1.24 ± 1.30	1.22 ± 0.99	1.27 ± 0.98	0.358	0.63 ± 1.29	0.083
**Height** *(cm)*	127.37 ± 10.41	128.98 ± 10.91	124.96 ± 9.29	0.064	123.75 ± 10.56	0.208
**Height-SDS**	1.00 ± 0.94	1.21 ± 0.99	0.70 ± 0.77	**0.016**	0.29 ± 0.91	**0.006**
**BMI** *(kg/m*^*2*^*)*	17.77 ± 2.29	17.46 ± 2.08	18.2 ± 2.54	0.090	17.28 ± 3.02	0.459
**BMI-SDS**	0.99 ± 1.01	0.85 ± 0.92	2.21 ± 1.10	0.080	0.59 ± 1.30	0.169
**Bone age** *(y)*	8.65 ± 1.99	9.20 ± 1.81	7.87 ± 2.00	**0.006**	7.28 ± 1.23	**0.025**
**Mid parental height** *(cm)*	162.39 ± 4.84	162.24 ± 3.72	162.62 ± 6.25	0.383	163.91 ± 3.93	0.240
**Predicted adult height** *(cm)*	161.11 ± 7.30	160.68 ± 6.11	161.77 ± 8.96	0.600	164.17 ± 7.23	0.194
**Tanner stage** *n (%)*						
II	41 (63%)	22	19			
III	20 (31%)	13	7	0.100	Prepubertal	NA
IV	4 (6%)	4	–			
**Gestational age** *n (%)*						
< 37 weeks	6 (9%)	5 (13%)	1 (4%)	0.221	1 (1%)	0.135
37–41 weeks	59 (91%)	34 (87%)	25 (96%)		16 (9%)	
**Birth weight** *n (%)*						
< 2500 g	6 (9%)	3 (9%)	3 (12%)		1 (1%)	
2501–4000 g	57 (88%)	35 (90%)	22 (85%)	0.827	16 (9%)	0.683
> 4001 g	2 (3%)	1 (1%)	1 (3%)		-	
**Positive familiar history PP** *n (%)*	7 (11%)	3 (8%)	4 (15%)	0.424	1 (1%)	0.545

The patient cohort included 11 individuals (16%) from Brazil, African countries, and Asia. The control group included one child from Brazil. In the entire sample, one adopted child (6%) was included.

On the basis of medical records and clinical and radiographic exams, only height-SDS (1.21 ± 0.99 versus 0.70 ± 0.77) and bone age (BA) (9.20 ± 1.81 vs 7.87 ± 2.00) were significantly greater among girls with progressive CPP and NP-CPP (*p* = 0.016 and *p* = 0.006, respectively). The differences remained similar when we compared children with pubertal signs and controls (height SDS 1.00 ± 0.94 vs 0.29 ± 0.91; *p* = 0.006; BA 8.65 ± 1.99 vs 7.28 ± 1.23; *p* = 0.025).

Hormonal and metabolic profiles were analysed in all girls with pubertal signs, and every basal profile was greater in those with progressive CPP (Table 2). Basal LH is not measurable in many patients, even those with progressive CPP (11, 28%), so median values are not very reliable. As the basal LH/FSH ratio is a composite value, it has the same limitation.

**Table 2:** Hormonal profiles of the progressive CPP group and nonprogressive CPP group

	Progressive CPP	NP-CPP	*p value*
	*n* = 39	*n* = 26	
**IGF1** (ng/ml)	270.15 ± 78.48	174.12 ± 48.97	** < 0.001**
**IGF1-SDS**	1.94 ± 0.80	0.79 ± 0.66	** < 0.001**
**Basal FSH** (mUI/mL)	3.78 ± 2.06	1.72 ± 1.03	** < 0.001**
**Basal LH** (mUI/mL)*	0.98 (0.10–2.70)	0.0 ± 0.0	** < 0.001**
Basal ratio LH/FSH*	0.22 (0.48–0.63)	0.0 (0.0–0.1)	** < 0.001**
**AMH** (pmol/L)*	18.4 (10.05–26.13)	14.2 (11.65–22.85)	0.801
**Estradiol** *n* (%)^+^	32 (74%)	11 (42%)	** < 0.001**

^*^Mann‒Whitney test

^+^Chi‒square test

Pelvic ultrasound evaluation revealed that only 31 of the 39 (79%) girls who met the CPP criteria were pubertal.

**Fig. 2:**
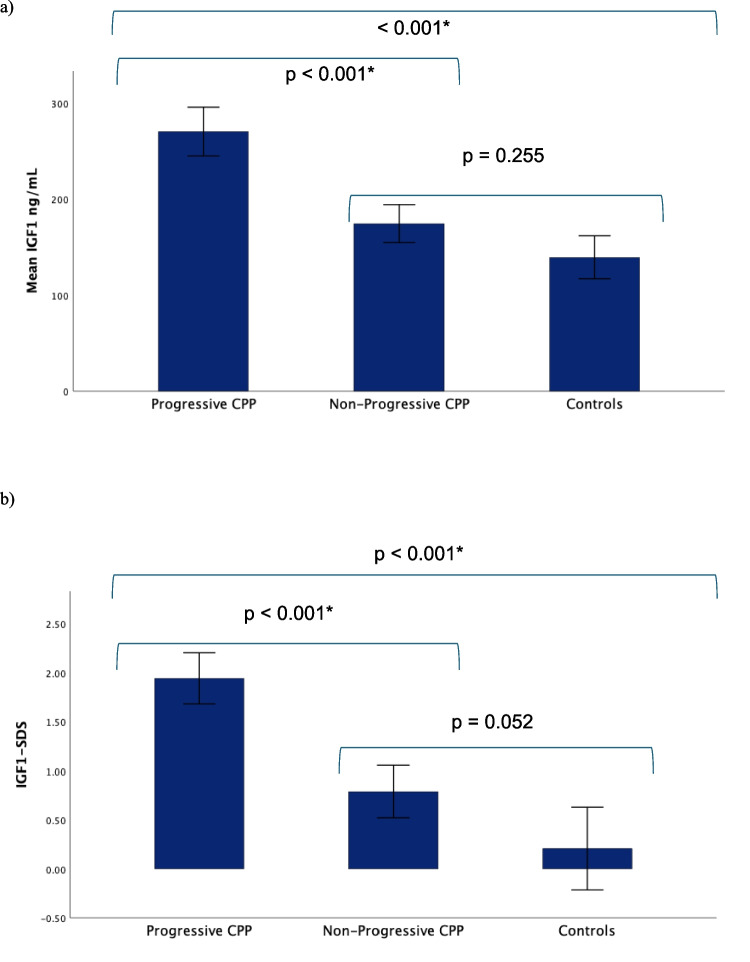
**a** IGF1 (mg/dL) distribution and comparisons among groups. **b** IGF1‒SDS distribution and comparisons among groups

The mean basal IGF1 and IGF1-SDS levels were significantly greater in progressive CPP patients (IGF1 270.15 ng/mL ± 12.567; IGF1-SDS 1.943 ± 0.129) than in both NP-CPP patients (IGF1 174.12 ng/mL ± 9.604; IGF1-SDS 0.788 ± 0.129) and controls (139.28 ng/mL ± 10.465; IGF1-SDS 0.208 ± 0.1995). The mean IGF1 and IGF1-SDS values were not significantly different between the NP-CP group and the control group (*p* = 0.255 and *p* = 0.052, respectively), as shown in Fig. 2.

A multivariate logistic regression was performed to account for potential confounders, which revealed that both IGF1 (unadjusted OR 1.024) and IGF1-SDS (unadjusted OR 7.320) remained statistically significant (*p* < 0.001). The adjusted odds ratios (ORs) were 1.025 for IGF1 (95% CI 1.010–1.040) and 8.721 for IGF1–SDS (95% CI 2.624–28.986).

**Fig. 3:**
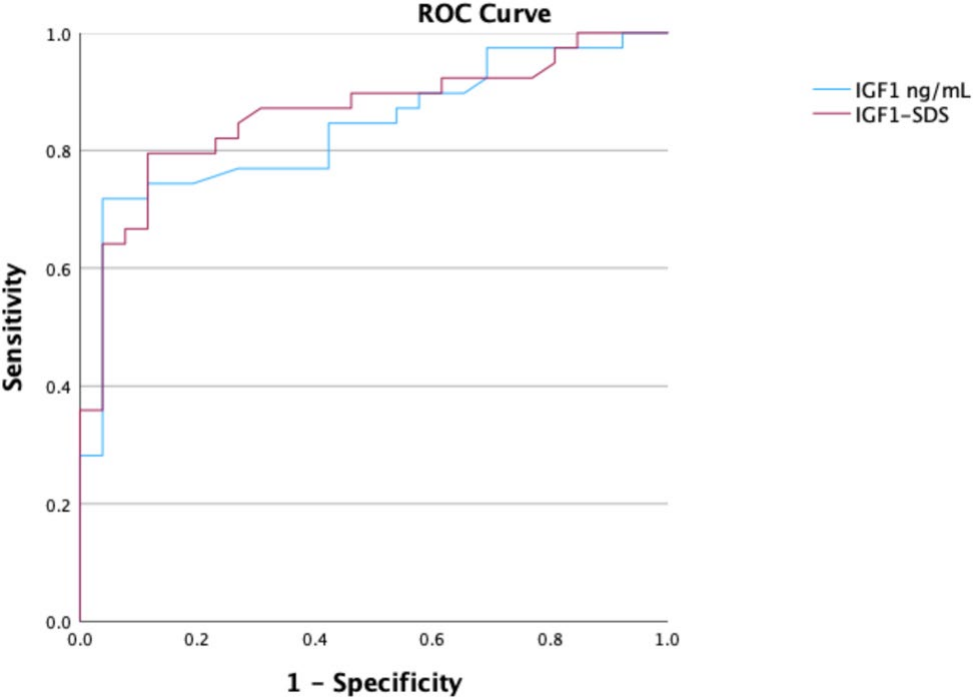
Receiver operating characteristic curves for IGF1 and IGF1-SDS

The AUC of IGF1 was 0.837 (95% CI 0.738–0.935), and that of IGF1–SDS was 0.862 (95% CI 0.771–0.953) (Fig. 3).

In our study, a mean IGF1 value of 231.0 ng/mL had 71.8% sensitivity and 97.7% specificity, with a Youden index of 0.680. The cut-off IGF1-SDS value of 1.44 had 79.5% sensitivity, 90.7% specificity, and a Youden index of 0.702. The overall accuracy of the mean IGF1 values was 82.2%, whereas that of the IGF1-SDS was 77.8%, as shown in Table 3.

**Table 3:** Diagnostic performance of IGF1 and IGF1-SDS for progressive CPP

	**AUC** (95%CI)	**Cut-off values**	**Sensitivity** (%)	**Specificity** (%)	**Accuracy** (%)	**Youden Index**	*p value*
**IGF1** (ng/mL)	0.837 (0.738–0.935)	**231**	71.8	97.7	82.2	0.680	** < 0.001**
**IGF1-SDS**	0.862 (0.771–0.953)	**1.44**	79.5	90.7	77.8	0.702	** < 0.001**

## Discussion

This study revealed that mean serum IGF1 levels and IGF1-SDS are greater in CPP girls than in prepubertal controls. Compared with nonprogressive CPP, progressive CPP is associated with significantly greater IGF1 and IGF1-SDS levels or isolated thelarche, with cut-offs of 231 ng/mL for IGF1 and 1.44 ng/mL for IGF1-SDS.

No significant differences were found between nonprogressive CPP patients and controls, although the IGF1 values were intermediate. This fact is fundamental when we intend to propose both serum IGF1 and IGF1-SDS as diagnostic tools to decide whether to proceed to the GnRH stimulation test.

On the basis of clinical signs, auxological measurements, ultrasound assessments of pubertal development, and bone age, we were not able to differentiate between CPP and NP-CPP.

In our study, even after adjusting for height-SDS and bone age, increased levels of IGF1 and IGF1-SDS were associated with increased odds of having progressive forms of CPP. Age was adjusted by using SDS values, and consistent with other studies, BMI and BMI-SDS had no impact on serum IGF1 levels [13, 31].

The link between growth and the puberty axis has been previously studied. Our finding of elevated IGF1 levels in CPP girls compared with controls aligns with studies by Sørensen et al. [31], Juul et al. [32], and Chang et al. [24], which also reported higher IGF1 levels in girls with CPP than in their age-matched healthy peers. Notably, since 1989, Fontoura et al. [33] highlighted the role of IGF1 levels in distinguishing slowly and rapidly progressing variants of CPP. Since then, most researchers have focused on exploring the role of IGF1 in triggering puberty rather than its diagnostic value [34, 35]. Other studies, such as Muratoglu et al. [36], emphasize the importance of monitoring IGF1 levels during treatment with GnRH analogues. Sales et al. [37] revisited the differences in IGF1 levels between isolated premature thelarche (IT) and CPP, concluding that IGF1 values were intermediate between those of prepubertal children and CPP. This suggests that it may represent an early stage of puberty with subtle yet real changes in the GH‒IGF axis. These findings are identical to our findings, although neither study demonstrated statistical significance.

In 2021, Escagedo et al. [13] introduced the importance of evaluating IGF1-SDS to distinguish between progressive forms of CPP and isolated premature adrenarche/precocious thelarche in girls aged 6–8 years whenever the initial clinical and laboratory approach was inconclusive. Their retrospective study faced a relevant limitation due to changes in IGF1 assays during the evaluation period, which they addressed by using IGF1-SDS instead of mean IGF1 levels. In their analysis of the diagnostic value of IGF1-SDS for differentiating CPP from normal pubertal variants such as IT, they proposed an IGF1-SDS cut-off value of 1.75, with a specificity of 94%. These values are very close to our cut-off of 1.71, with a specificity of 96.2%.

Recently, in 2024, Wang et al. [38] proposed principal component analysis for the differentiation of idiopathic central precocious puberty from premature thelarche. In their mathematical procedure, they include IGF1-SDS because it is one of the biochemical parameters that has significantly higher values in CPP girls. In addition to being one large study (143 idiopathic CPP girls versus 91 premature thelarche girls), this study is retrospective and uses two different assays to determine IGF1 (IMMULITE Siemens 2000™ before 2013 and IDS-iSYS™ after), explaining why they only use IGF1-SDS values to minimize bias.

The concern about different IGF1 assays is a major limitation, particularly given that concordances between the manufacturers’ reference intervals and those obtained in real-world healthy adult populations are generally poor [39, 40].

From our findings and comparing the diagnostic performance of the proposed cut-off values, IGF1-SDS demonstrated a higher sensitivity, AUC, and Youden index. Additionally, using an IGF1-SDS cut-off offers further advantages, such as correction for the age-related increase in absolute IGF1 values and allows broader applicability across different assays [13, 19, 38]. In our study, absolute IGF1cut-off value has a specificity of 97.7%, but the lower sensitivity compared to IGF1-SDS makes the later more reliable biomarker. Absolute IGF1 values still have a role, particularly in day-to-day clinical practice by general practitioners and paediatricians who may not be familiar with IGF1-SDS calculation, especially until laboratory reports routinely include IGF1-SDS values.

To our knowledge, this is the first prospective study to evaluate the diagnostic value of serum IGF1 levels and IGF1-SDS during the initial assessment of girls presenting with early sexual development. Furthermore, we are the first to propose specific cut-off values for these markers, which are grounded in current evidence and demonstrate high specificity and strong accuracy. The cut-off definitions we use are for IMMULITE assays, which are among the most commonly assays used abroad [39, 40]. T

We suggest that a girl younger than 8 years of age, with breast development, advanced bone age, undetectable basal LH, and IGF1-SDS greater than 1.44, may not require a GnRH stimulation test. This approach could streamline the diagnostic process and reduce unnecessary invasive procedures.

One of the major strengths of our study is its prospective design, which includes a healthy control group and participants from two different centres. Importantly, the same blinded clinician conducted the Tanner evaluation, using consistent equipment for anthropometric measurements and identical laboratory assays across all participants. The major study limitation is the sample size, although it is large enough to show significant differences and is equivalent to other studies, with a retrospective or cross-sectional design [13, 15].

## Conclusions

Both the serum IGF1 level and the IGF1-SDS are significantly higher in girls with CPP than in age-matched controls and other girls with signs of puberty without progressive forms. IGF1-SDS demonstrates superior diagnostic performance, offering the advantages of age adjustment and greater comparability across different assays. Due to its high specificity, values above 1.44 SDS may significantly increase the posttest probability of CPP, avoiding invasive, stressful, and costly procedures such as GnRH stimulation tests. Therefore, IGF1-SDS, but also IGF1 absolute determination, is highly valuable in the initial laboratory evaluation of girls with early sexual development and should be incorporated into the CPP diagnostic procedure.

## Data Availability

Data is provided within the manuscript.

## Abbreviations

AUC: Area under the curve
AMH: Anti-Müllerian Hormone
BA: Bone age
BMI: Body mass index
CPP: Central precocious puberty
CI: Confidence interval
FSH: Follicle-stimulating hormone
GH: Growth hormone
GnRH: Gonadotropin-releasing hormone
HPG: Hypothalamic–pituitary–gonadal (axis)
IGF1: Insulin-like growth factor 1
IGF1-SDS: Insulin-like growth factor 1 standard deviation score
IT: Isolated Thelarche
LH: Luteinizing hormone
LHRH: Luteinizing hormone-releasing hormone
NP-CPP: Nonprogressive central precocious puberty
OR: Odds ratio
ROC: Receiver operating characteristic
SDS: Standard deviation score
STARD: Standards for Reporting Diagnostic Accuracy
STROBE: Strengthening the Reporting of Observational Studies in Epidemiology
WHO: World Health Organization
